# Nimotuzumab enhances temozolomide‐induced growth suppression of glioma cells expressing mutant EGFR in vivo

**DOI:** 10.1002/cam4.614

**Published:** 2016-01-18

**Authors:** Yusuke Nitta, Saki Shimizu, Yukiko Shishido‐Hara, Kaori Suzuki, Yoshiaki Shiokawa, Motoo Nagane

**Affiliations:** ^1^ Department of NeurosurgeryKugayama Hospital2‐14‐20 KitakarasuyamaSetagayaTokyo157‐0061Japan; ^2^ Department of NeurosurgeryKyorin University Faculty of Medicine6‐20‐2 ShinkawaMitakaTokyo181‐8611Japan; ^3^ Department of PathologyKyorin University Faculty of Medicine6‐20‐2 ShinkawaMitakaTokyo181‐8611Japan; ^4^ Department of Anatomic PathologyTokyo Medical University6‐7‐1 Nishi‐ShinjukuShinjukuTokyo160‐0023Japan

**Keywords:** Drug resistance, EGFRvIII, glioblastoma, MSH6, nimotuzumab, temozolomide

## Abstract

A mutant form of epidermal growth factor receptor (EGFR), EGFRvIII, is common in glioblastoma (GBM) and confers enhanced tumorigenic activity and drug resistance. Nimotuzumab, an anti‐EGFR antibody, has shown preclinical and clinical activity to GBM, but its specific activity against EGFRvIII has not been fully investigated. Human glioma U87MG or LNZ308 cells overexpressing either wild‐type (wt) EGFR or EGFRvIII were treated with nimotuzumab, temozolomide, or both. Expression and phosphorylation status of molecules were determined by Western blot analysis. Methylation status of promoter region of *O* ^*6*^‐methylguanine‐DNA methyltransferase (*MGMT*) was detected by methylation‐specific PCR. Antitumor activity was tested using nude mice bearing either subcutaneous or intracerebral xenografts along with analyses of EGFR phosphorylation status, proliferation, apoptosis, and vessel density. Nimotuzumab treatment resulted in reduction of EGFRvIII tyrosine phosphorylation with a decrease in Akt phosphorylation that was greater than that of wtEGFR. Correspondingly, antitumor effects, growth suppression and survival elongation, were more significant in mice bearing either subcutaneous or intracerebral tumor expressing EGFRvIII than in those expressing wtEGFR. These effects were markedly increased when temozolomide was combined with nimotuzumab. The post‐treatment recurrent brain tumors exhibited a decrease in expression of the mismatch repair (MMR) proteins, MSH6 and MLH1, but their methylated *MGMT* status did not changed. Nimotuzumab has in vivo antitumor activity against GBM, especially those expressing EGFRvIII, when combined with temozolomide. This could provide a basis for preselection of patients with GBM by EGFR status who might benefit from the nimotuzumab and temozolomide combination therapy.

## Introduction

Glioblastoma (GBM) is the most frequent primary brain tumor that remains incurable despite multimodal treatments available. Prognosis of patients with GBM had been dismal with median overall survival being <15 months when treated with the standard of care which is temozolomide (TMZ) and radiotherapy (RT) 1. As chemotherapy has only a marginal survival benefit and is usually toxic to patients with GBM, development of novel therapeutic strategies which could specifically target GBM cells but not normal cells remains an urgent issue for enhancing efficacy and reducing toxicity.

Recent comprehensive genomic analysis on GBM by The Cancer Genome Atlas Research Network has identified three major signaling pathways that are altered at high frequencies (80 ~ 90%) among GBMs 2. The most frequent genetic alteration associated with GBM is amplification of the epidermal growth factor receptor (EGFR) gene, which results in overexpression of the EGFR. This overexpression promotes cell proliferation, angiogenesis and is an indicator of bad prognosis. It is known that half of *EGFR* gene amplifications include rearrangements 3, the most common being a genomic deletion of exons 2–7, resulting in an in‐frame deletion variant that has a truncated extracellular domain with ligand‐independent constitutive activity (EGFRvIII, also called ∆EGFR) 3.

EGFRvIII is expressed in tumors in about 30% of GBM patients. EGFRvIII is a functional and permanently activated mutation of the EGFR, a protein that contributes to cell growth and has been well validated as a target for cancer therapy. Unlike EGFR, EGFRvIII has not been detected at a significant level in normal tissues, but has been identified in GBM. Cells producing EGFRvIII have an enhanced capacity for unregulated growth and are associated with more aggressive disease and worse prognosis 4.

Overexpression of EGFRvIII in human GBM cells results in constitutively active receptor expression and enhances tumorigenicity in nude mice, to a significantly greater extent than overexpression of wild‐type (wt) EGFR 5, 6, which is mediated by both an increase in proliferation and a decrease in apoptosis of tumor cells 7, as well as inducing angiogenesis and conferring chemoresistance that is associated with upregulation of anti‐apoptotic Bcl‐X_L_ 7, 8, 9. Inhibition of EGFRvIII by a tyrosine kinase inhibitor AG1478 downregulates Bcl‐X_L_ expression and restores sensitivity of GBM cells to cisplatin 9, and this combination treatment suppresses growth of GBM xenografts overexpressing EGFRvIII 10, suggesting that targeting EGFR signaling could be a reasonable approach for high‐grade glioma therapy.

Several agents, including small molecule tyrosine kinase inhibitors (TKIs) and monoclonal antibodies (mAbs) specific for EGFR, have been designed to selectively block EGFR signaling. Phase II studies of gefitinib or erlotinib single treatments for patients with recurrent GBM have shown minimal overall efficacy with median progression‐free survival from 2 to 3 months, although some responders were obtained 11. Various mAbs to EGFR are also under investigation for their efficacy as anticancer agents. An anti‐EGFR mAb, cetuximab, which had shown to be efficacious in head and neck cancer 12, was able to decrease proliferation and to increase apoptosis in highly EGFR‐overexpressing human GBM xenografts 13. The antibody‐based approach has also been tested in Phase I/II clinical trials for patients with GBM with or without combination of other cytotoxic agents or radiotherapy 14. Nimotuzumab (also known as h‐R3) is a humanized anti‐EGFR mAb, which is currently on clinical evaluation. It binds to an epitope located in extracellular domain III of EGFR, which is preserved in the mutant EGFRvIII 15. In preclinical studies, nimotuzumab showed antiproliferative, proapoptotic, and antiangiogenic effects in tumors that overexpress EGFR 16, and enhanced radiosensitivity with reduction of tumor blood vessels and proliferation in the human GBM cell line U87MG 17. Nimotuzumab had shown enhanced sensitivity associated with reduction of phosphorylated Akt either as single agent or in combination with rapamycin when it was used on U87MG GBM cells and on patient‐derived glioma cell NN137 that expressed EGFRvIII. Moreover, nimotuzumab and rapamycin markedly reduced the percentage of viable cells in EGFR‐null cells which are resistant to rapamycin alone (Chong QD et al. personal communication).

Nimotuzumab has shown clinical benefit when is used as monotherapy in recurrent or relapsed brain tumors in children, or in combination with RT in children with diffuse intrinsic pontine glioma (DIPG) or in adult with GBM, and this antitumor activity was observed in the absence of severe skin rash, acneiform rash, and any other dermatological or mucosal toxicities commonly associated with other EGFR‐targeting agents, such as cetuximab, panitumumab, and erlotinib 18, 19, 20, 21, 22.

The use of EGFR specific antibodies for cancer therapy is promising, but serious problems can be envisioned, due to heterogeneity within the tumor and the fact that normal cell also expresses EGFR. Therefore, it will be preferable to target cancer cells with antibodies that recognize a target that is expressed on tumor cells to avoid toxicity in normal cells. In this regards, EGFRvIII is particularly interesting as the deletion in the extracellular domain creates a tumor‐specific epitope that can be used as a target for anti‐EGFR antibodies for targeting the fastest proliferating cells. As little is known about the antitumor efficacy and resistance of nimotuzumab in combination with TMZ in those GBM‐overexpressing EGFRvIII or wtEGFR, our purpose will be to investigate in vivo the growth‐inhibitory effects of nimotuzumab alone and in combination with TMZ in GBM cell lines that overexpress EGFR species and the changes in molecular determinants studying the mechanisms for sensitivity of tumor cells that had evaded the nimotuzumab/TMZ combination treatment.

## Materials and Methods

### Regents

Nimotuzumab was provided by Daiichi Sankyo Co. Ltd. (Tokyo, Japan) Temozolomide was purchased from LKT Laboratories (St. Paul, MN) and dissolved in dimethyl sulfoxide (DMSO) at stock concentration of 100 mM. Tyrphostin AG1478 was obtained from Wako (Osaka, Japan) and dissolved in DMSO at stock concentration of 100 mM.

### Cell lines and culture conditions

The human GBM cell line U87MG, which expresses trace levels of wtEGFR, and its sublines, U87MG.∆EGFR and U87MG.wtEGFR which overexpress EGFRvIII and exogenous wtEGFR, respectively, were described previously 6. Similarly, the human GBM cell line LNZ308 and its sublines, LNZ308.∆EGFR and LNZ308.wtEGFR, were described previously 23. All cells were cultured as described 7. Treatment‐resistant subpopulations of U87MG.∆EGFR line, designated as U87MG.∆EGFR escaper, were generated by reculturing resected xenografts in dish that had eventually regrown in nude mice after in vivo treatments indicated.

### In vitro cell proliferation assay

Cells were plated onto 12‐well plates at 1 × 10^5^ cells/well in the fully supplemented medium for overnight and were then treated with either AG1478 (15 *μ*M), nimotuzumab (0.25–4 *μ*M), TMZ (20 *μ*M), or in combination with nimotuzumab and TMZ for 24 to 72 h, when cells were lysed for further analysis.

### Western blotting

Whole cell lysates were prepared in RIPA buffer and were subjected to Western blot analyses as previously described 7. Proteins on the PVDF membranes were probed with antibodies against EGFR (C13, BD Biosciences, San Jose, CA), P‐EGFR (Tyr1068), P‐EGFR (Tyr1173), Akt, P‐Akt (Thr308), P‐Akt (Ser473), P‐mammalian target of rapamycin (mTOR) (Ser2448), mTOR (Cell Signaling, Danvers, MA), *O* ^*6*^‐methylguanine‐DNA methyltransferase (MGMT) (MT3.1, Neo Markers, Fremont, CA), MSH6, MLH1, MSH2, PMS2 (BD Biosciences), detected by chemiluminescence, and quantified (ImageQuant LAS4010, GE Healthcare, Tokyo, Japan). Loading of lysates on membranes was evaluated by *β*‐actin blot or Ponceau S staining (St. Louis, MO, Sigma‐Aldrich).

### In vivo experiments

Human GBM cells (2 × 10^6^ cells) were suspended in 0.1 mL PBS and injected subcutaneously into the right flank of 4‐ to 5‐week‐old female nude mice of BALB/CA (Saitama Experimental Animals Supply, Co. Ltd., Saitama, Japan). Mice were treated when tumor volume was 100 ~ 300 mm^3^. For intracerebral stereotactic inoculation, 5 × 10^5^ GBM cells in 5 *μ*L of PBS were inoculated into the right corpus striatum of the mouse brain as described 7. Treatment groups consisted of vehicle control (V), nimotuzumab alone (N), TMZ alone (T), and the combination of nimotuzumab and TMZ (NT). Nimotuzumab was administered i.p. in a single dose of 1.0 mg/mouse, three times per week, for three consecutive weeks per treatment cycle; mice in the vehicle and TMZ alone groups were injected with normal saline. TMZ was also administered i.p. in a single dose of 0.1 mg/mouse for three consecutive days from the start day of the cycle; mice in the vehicle and nimotuzumab alone groups were injected with DMSO. The growth of tumors was measured as described 10. Systemic toxicity of the treatments was assessed by change in body weight and by organ inspection at autopsy. Mice were sacrificed by CO_2_ inhalation when they became moribund. All animal procedures were approved by The Experimental Animal Ethics Committee in Kyorin University.

### In vivo proliferation assay

Cross sections of formalin‐fixed paraffin‐embedded xenografts in nude mice were stained immunohistochemically with anti‐human MIB‐1 antibody (1:100 dilution) (DAKO, Tokyo, Japan) as described 10. The percentage of tumor nuclei reactive to MIB‐1 antibody (labeling index; LI) was estimated following examination of five high‐power fields using the software Gunma LI 24. An average of 1500 cells were examined and scored in areas of the tumor judged to be most representative.

### Apoptosis assay

Apoptotic cell death was determined by TUNEL assays using In Situ Cell Death Detection Kits (Roche) as described 7. The apoptotic index (%) was calculated as the ratio of apoptotic cell number to total tumor cell number after examination of more than 1000 cells.

### Vessel density evaluation

To assess vessel density in brain tumors, cross sections of formalin‐fixed paraffin‐embedded tumors were stained immunohistochemically with a rabbit polyclonal anti‐CD31 antibody (ab28364, Abcam; 1:50) as described 10. Vessel density was analyzed by counting a number of CD31‐stained vessels per a high‐power field (×200 magnification), and the vessel number was calculated as the mean of counts in 10 fields per section randomly selected from non‐necrotic areas of the tumors.

### Methylation‐specific PCR

Methylation status of the *MGMT* gene promoter was determined by methylation‐specific PCR using the EZ DNA Methylation Kit (ZYMO RESEARCH CORP, Irvine, CA) as described 25. Briefly, DNAs were subjected to bisulfite treatment that modifies unmethylated, but not methylated, cytosines to uracil. After purification, DNA was amplified using specific primers for the methylated (*M*) and modified unmethylated *MGMT* promoter (*U*), respectively. Normal peripheral blood leukocyte DNA (NC) was negative for methylation, whereas human genomic DNA with enzymatic methylation (PC) was used as a positive control for methylation. The PCR products were separated on a 2.7% agarose gel. The presence of a PCR product in the *U Lane* (93 bp) signifies the presence of unmethylated *MGMT* promoter, whereas a PCR product in the *M Lane* (81 bp) indicates the presence of methylated promoter.

### Statistical analysis

The data were analyzed for significance by Student's *t*‐test. Survival of mice bearing intracerebral xenografts was calculated according to the Kaplan–Meier method, and differences in survival were evaluated with the log‐rank test. All statistical analyses were carried out using the statistical package SPSS version 20 (IBM, Armonk, NY).

## Results

### Effect of nimotuzumab on EGFR phosphorylation

It was determined in vitro whether the anti‐EGFR mAb nimotuzumab could suppress phosphorylation of EGFR in human GBM cells overexpressing either EGFRvIII or wtEGFR and the well‐characterized human glioma cell lines U87MG.∆EGFR and U87MG.wtEGFR were used on the experiments 6, 7. In these cells, both EGFRvIII and wtEGFR were phosphorylated under the standard 10% serum containing on the culture conditions. Phosphorylated EGFRvIII was visualized only on the cell line U87MG.∆EGFR at 150 kDa, whereas phosphorylated wtEGFR was detectable only in U87MG.wtEGFR cells at 170 kDa.

**Figure 1:**
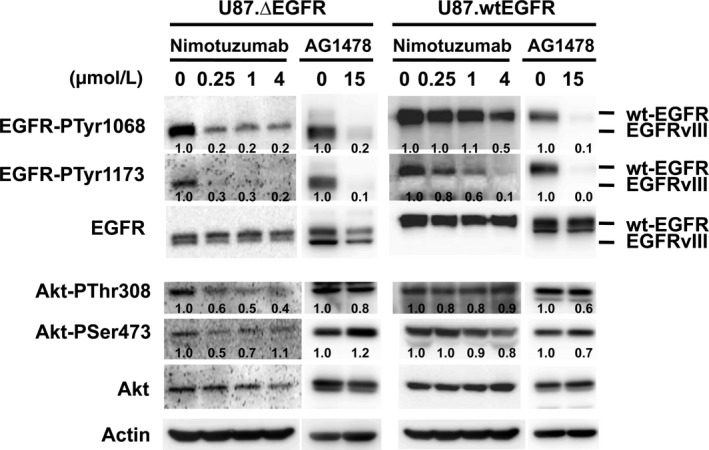
Dephosphorylation of EGFRvIII (∆EGFR) and wild‐type (wt) EGFR upon treatment with nimotuzumab or tyrphostin AG1478 in vitro. Cultured human glioma U87MG cells overexpressing either EGFRvIII (U87MG.∆EGFR) or wtEGFR (U87MG.wtEGFR) were treated with nimotuzumab for 72 h and their lysates were subjected to Western blot analysis. Nimotuzumab dephosphorylated EGFR at both tyrosine residues 1068 and 1173. EGFRvIII tyrosine phosphorylation was preferentially suppressed by nimotuzumab at lower doses, compared with wild‐type EGFR. Akt phosphorylation at threonine residue 308 was modestly suppressed in U87MG.∆EGFR cells by nimotuzumab. Relative tyrosine phosphorylation per molecule is shown below each lane, calculated as a ratio of that of untreated status and standardized with actin expression. A tyrphostin AG1478 was used as a positive control for EGFR inhibition.

**Figure 2:**
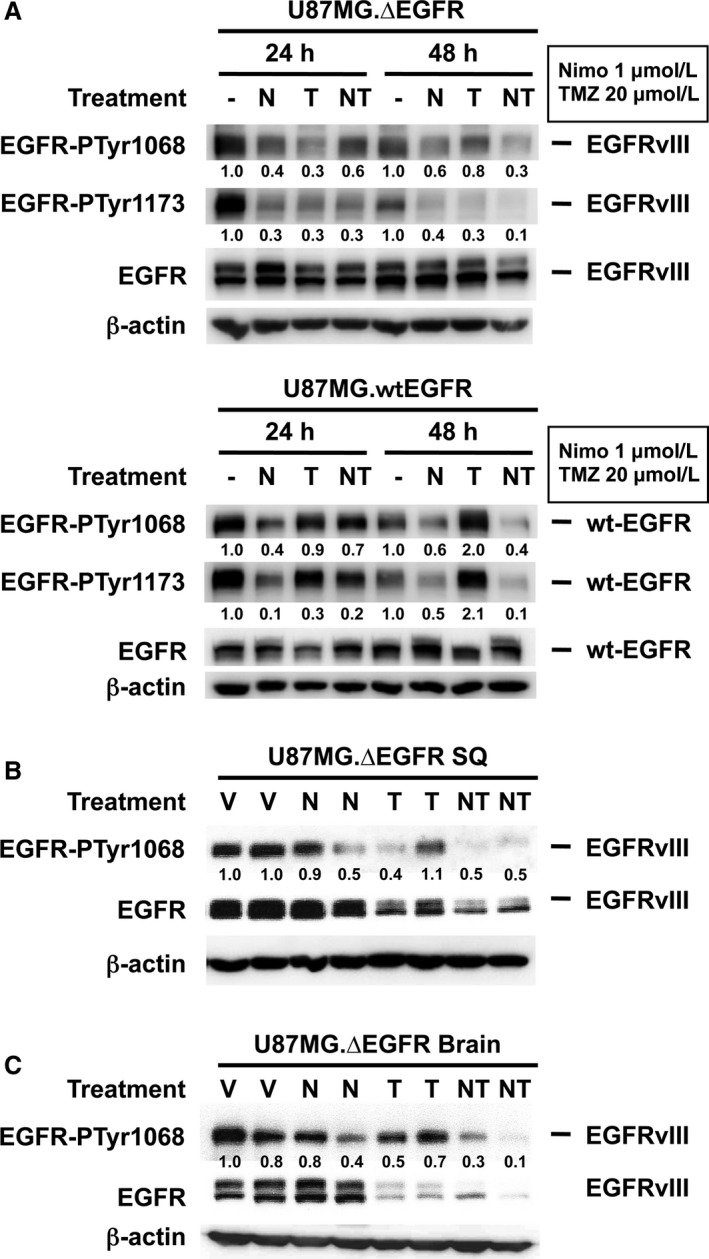
Suppression of EGFRvIII tyrosine phosphorylation by combination treatment with nimotuzumab and temozolomide (TMZ) in U87MG.∆EGFR in vitro (A) and in vivo (B, C). A. Cultured U87MG.∆EGFR and U87MG.wtEGFR cells were treated with nimotuzumab and TMZ in vitro, and their total cell lysates were subjected to Western blot analysis. Treatments are as follows: ‐, DMSO control; N, nimotuzumab 1 *μ*M; T, TMZ 20 *μ*M; NT, nimotuzumab; and TMZ combination. B, C. Dephosphorylation of EGFRvIII in mice subcutaneous (B) or intracerebral (C) xenografts upon treatments with either vehicles (V), nimotuzumab alone (N), TMZ alone (T), or both (NT) as described in “Materials and Methods.” Relative tyrosine phosphorylation per molecule is shown below each lane and calculated as a ratio of that of either untreated or vehicle‐treated status and is also standardized with actin expression.

Nimotuzumab treatment in these cells exhibited reduction of tyrosine phosphorylation of both EGFRvIII and wtEGFR determined by Western blot analysis using EGFR phosphotyrosine‐specific antibodies. The extent of tyrosine dephosphorylation of EGFR, was much weaker than the one elicited by the EGFR‐selective tyrphostin, AG1478 (Fig. 1) 9. Nimotuzumab suppressed tyrosine phosphorylation at both residues 1068 and 1173 of the EGFRvIII C‐terminal tail at doses as low as at 0.25 *μ*M and also dephosphorylated wtEGFR at tyrosine residue 1173 at a dose of 0.25 *μ*M, but required a dose of 1 to 4 *μ*M for tyrosine residue 1068 (Fig. 1, 2A), suggesting a differential anti‐EGFR activity depending on EGFR conformation.

To explore the effects of nimotuzumab on signaling downstream molecules of EGFR, the phosphorylation status of Akt and mTOR that are major effectors of EGFR signaling was tested (Fig. 1). Although AG1478 did not effectively dephosphorylate Akt in U87MG.∆EGFR cells, nimotuzumab treatment at 0.25 *μ*M resulted in a modest reduction in Akt phosphorylation at threonine codon 308, which was minimum in U87MG.wtEGFR cells. In contrast, nimotuzumab did not consistently alter phosphorylation of either Akt serine 473, mTOR, or MAP kinases in either cell type (data not shown). These results suggest that unlike potent small molecule EGFR inhibitors such as AG1478, suppression of EGFR activity and signaling by nimotuzumab may be partial and distinct, and that nimotuzumab might preferentially affect EGFRvIII signaling compared with wtEGFR.

### Reduction of EGFRvIII phosphorylation by combination treatment with nimotuzumab and TMZ in vitro and in vivo

We next assessed the EGFR status in vitro after TMZ treatment with or without nimotuzumab in U87MG.∆EGFR and U87G.wtEGFR cells. While TMZ (20 *μ*M) treatment moderately affects the tyrosine phosphorylation level of EGFRvIII to a greater extent than wtEGFR, addition of nimotuzumab (1 *μ*M) to TMZ led to marked reduction of the tyrosine phosphorylation at 48 h after treatment (Fig. 2A), although augmentation of TMZ‐induced cytotoxicity by nimotuzumab was less potent on in vitro cytotoxic or clonogenic assays (data not shown).

Nude mice bearing subcutaneous U87MG.∆EGFR xenografts were treated with nimotuzumab and/or TMZ, and were sacrificed after the first week of treatment (6 days after the start of treatment) to prepare tumor lysates to examine EGFR status of the tumor cells. Treatment with nimotuzumab alone and TMZ alone resulted in a slight reduction in EGFRvIII phosphorylation, whereas total EGFRvIII phosphorylation levels markedly decreased when combination of nimotuzumab and TMZ was used; this was also associated with a decrease in the EGFR expression level (Fig. 2B). Similarly, in intracerebral U87MG.∆EGFR xenografts, total EGFRvIII tyrosine phosphorylation was slightly reduced by nimotuzumab monotherapy treatment. In the TMZ‐treated tumors, the phosphorylation level per molecule did not change with a high level of total phosphorylation retained, while there was a decrease in the expression level of the receptor. Nimotuzumab and TMZ combination treatment resulted in marked decrease in both expression level and total tyrosine phosphorylation of EGFRvIII compared with the other treatments (Fig. 2C).

### Inhibition of EGFR signaling by nimotuzumab augments in vivo tumor growth suppression by TMZ

We then determined whether nimotuzumab could enhance the antitumor effects of TMZ, the most active, standard chemotherapeutic agent for GBM. Xenografts derived from GBM cells overexpressing EGFRvIII or wtEGFR, as well as parental cells in nude mice, were developed. Mice bearing established subcutaneous xenografts were treated systemically with either nimotuzumab alone (N), TMZ alone (T), both nimotuzumab and TMZ (NT), or vehicles (V) as described in “Materials and Methods.” None of the treatment regimens caused significant loss of body weight over the course of treatment.

**Figure 3:**
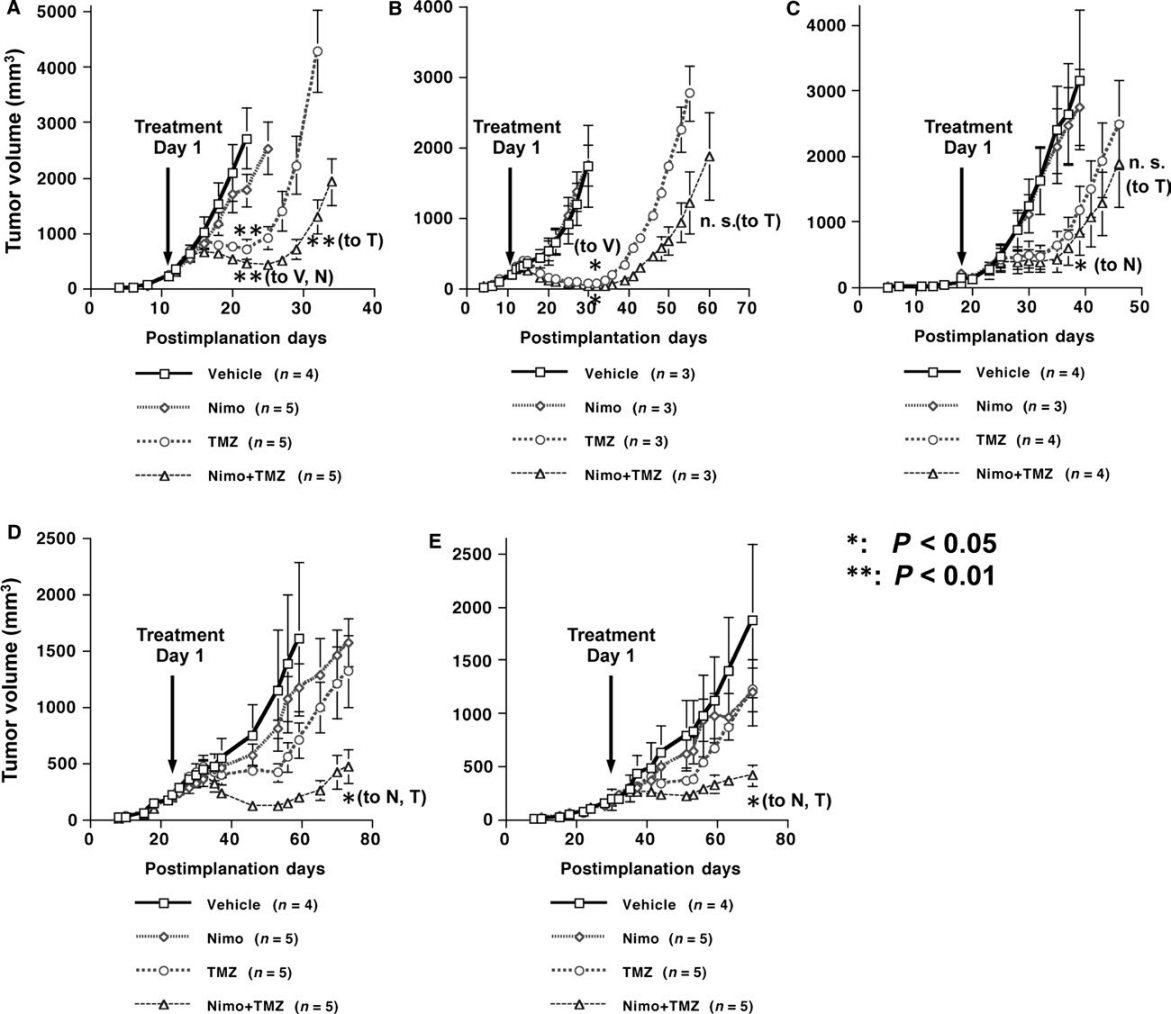
Growth inhibition of subcutaneous human glioma xenografts in BALB/CA mice upon treatment with nimotuzumab, temozolomide (TMZ), or both in vivo. Mice tumors derived from either U87MG.∆EGFR (A), U87MG.wtEGFR (B), U87MG (C), LNZ308.∆EGFR (D), or LNZ308.wtEGFR (E) were treated from the time point indicated (arrow) with the vehicle control (Vehicle or V), nimotuzumab (Nimo or N) (1.0 mg), TMZ (or T) (0.1 mg), or both (Nimo + TMZ; NT). **: *P* < 0.01; *: *P* < 0.05 (*t*‐test). Data are shown as the mean ± SE.

Nimotuzumab single treatment did not suppress the growth of subcutaneous tumors derived from any of U87MG.∆EGFR (Fig. 3A), U87MG.wtEGFR (Fig. 3B), or U87MG parental (Fig. 3C) cells. TMZ single treatment had a moderate antitumor activity in tumors of all U87MG cell types, compared with vehicle control or nimotuzumab single treatment (U87MG.∆EGFR: *P* < 0.01 to V, *P* < 0.05 to N on day 22; U87MG.wtEGFR: *P* < 0.05 to V on day 34; U87MG: *P* = 0.058 to N on day 39, Student's *t‐*test). Combination treatment with NT significantly delayed the growth of U87MG.∆EGFR xenografts (vs. T: 70% reduction in tumor volume, *P* < 0.01 on day 32) (Fig. 3A). In U87MG.wtEGFR tumors, a weaker tumor suppression was observed with NT combination treatment over TMZ alone, and this suppression did not reach statistical significance (*P* = 0.058) (Fig. 3B). In contrast, there was no significant additional growth retardation by the NT combination compared with TMZ alone in parental U87MG tumors expressing endogenous levels of EGFR (Fig. 3C). In another human glioma cell line LNZ308, similar enhanced growth suppression of subcutaneous xenografts derived from both LNZ308.∆EGFR (Fig. 3D) and LNZ308.wtEGFR (Fig. 3E) cells was observed by NT combination treatment over vehicle or nimotuzumab single treatment, to a slightly higher degree in EGFRvIII‐expressing tumors.

### Prolongation of survival of mice bearing intracranial xenografts by combination treatment with nimotuzumab and TMZ

**Figure 4:**
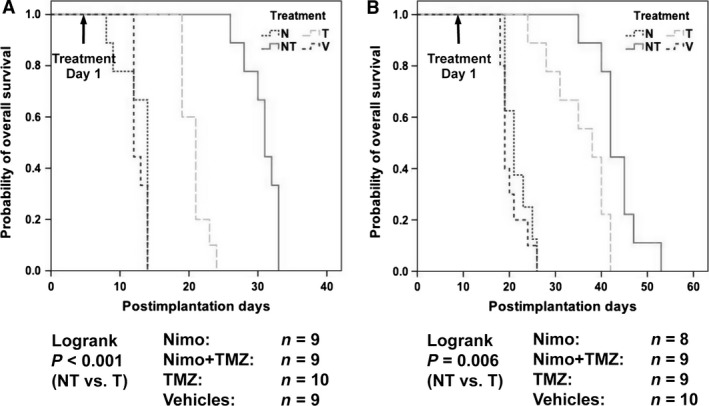
Survival elongation of BALB/CA mice bearing intracerebral xenografts derived from U87MG.∆EGFR (A) or U87MG.wtEGFR (B) by combination treatment with nimotuzumab and temozolomide (TMZ). Mice with established intracerebral tumors were treated from the time point indicated (arrow) as in Fig. 3. Mice were sacrificed when became moribund. Differences in survival were evaluated with the log‐rank test.

The potency of the combination treatment in intracerebral xenografts was examined. Consistent with the finding that NT combination treatment significantly inhibited EGFRvIII phosphorylation in intracerebral tumors (Fig. 2C), the combination treatment significantly extended the survival of mice bearing intracerebral xenografts compared to either vehicle, nimotuzumab, or TMZ treatment alone (*P* < 0.001 to T, log‐rank test). Nimotuzumab single treatment did not increase any survival benefit and TMZ had some limited activity compared with vehicle control treatment (Fig. 4A). In U87MG.wtEGFR intracerebral xenografts, similar antitumor activities were observed. Nimotuzumab had no effect, TMZ extended survival, and the combination exerted the best survival benefit among the treatments (*P* = 0.006 to T, log‐rank test) (Fig. 4B), although the survival gain over TMZ single treatment was smaller than that in mice with U87MG.∆EGFR xenografts. These results suggest that nimotuzumab could enhance TMZ‐mediated antitumor effects when combined, and its activity may be more potent in tumor cells overexpressing EGFRvIII than wtEGFR.

### Reduced proliferation, angiogenesis, and increased apoptosis of tumors upon nimotuzumab/TMZ combination treatment

**Figure 5:**
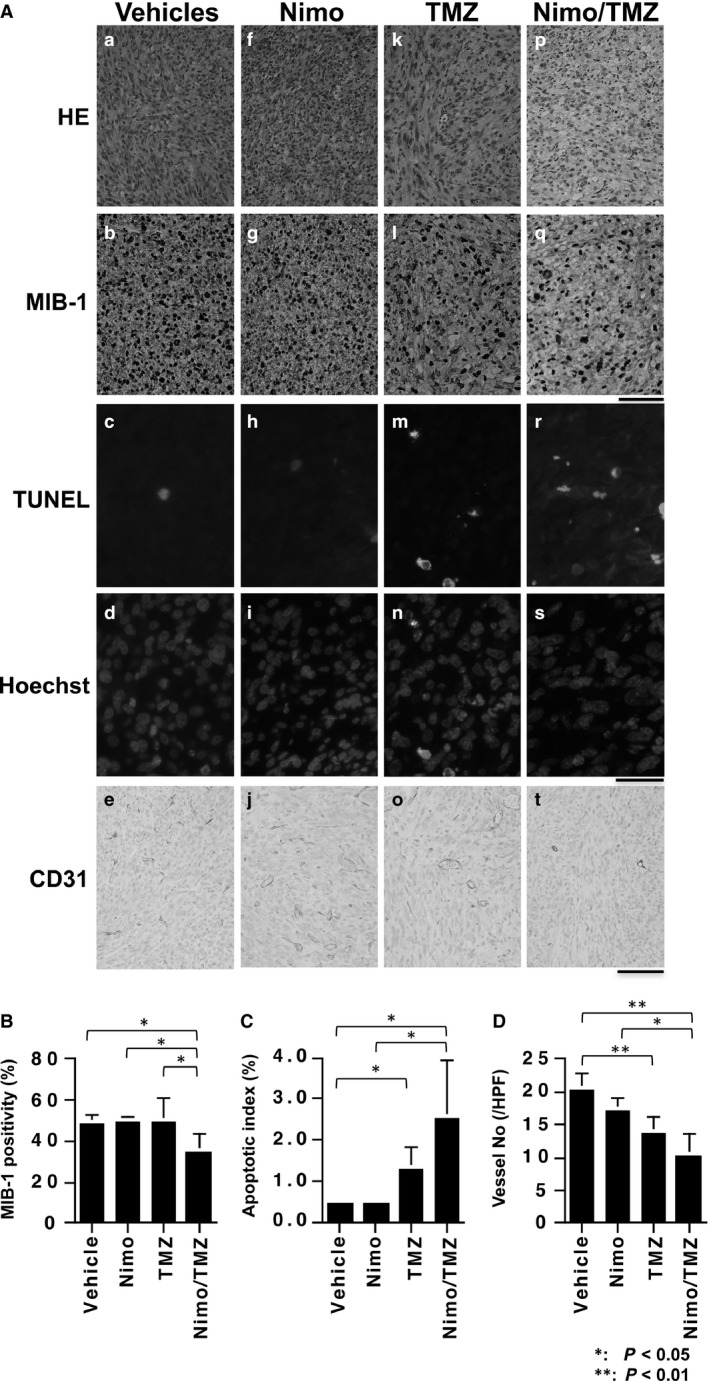
Combination treatment with nimotuzumab and temozolomide (TMZ) results in a reduction in proliferation and an increase in apoptosis in the tumor cells of the U87MG.∆EGFR xenografts. BALB/CA mice with intracerebral U87MG.∆EGFR xenografts were treated with either vehicles, nimotuzumab alone (Nimo), TMZ alone, or combination of nimotuzumab and TMZ (Nimo/TMZ) as described in Fig. 3. A. Microphotographs of paraffin‐embedded tumor sections with hematoxylin and eosin staining (HE) (top column), MIB‐1 staining (second column), TUNEL assay (third column) which was counterstained with Hoechst 33258 (fourth column), and CD31 immunostaining (bottom column). Bar, 100 *μ*m for HE, MIB‐1, and anti‐CD31 microphotographs; 50 *μ*m for TUNEL and Hoechst microphotographs. B. MIB‐1 positivity. MIB‐1‐positive cells and hematoxylin‐stained nuclei were counted. C. Apoptotic index. TUNEL‐positive cells (green nuclei) and Hoechst 33258‐stained nuclei (blue) were counted and apoptotic index (%) was calculated. D. Tumor vessel density. CD31‐stained tumor vessels were counted. **: *P* < 0.01; *: *P* < 0.05 (*t*‐test). Data are shown as the mean ± SD.

The rates of proliferation and apoptosis in treated intracerebral tumors were examined to determine the underlying mechanisms of tumor growth suppression by the NT combination treatment (Fig. 5A). After the first week of the treatment cycle, hypercellular tumor with brisk mitotic figures and capillary network was observed in control vehicle‐treated on intracerebral U87MG.∆EGFR xenografts (Fig. 5A‐a). NT combination treatment significantly suppressed proliferation (~30% reduction in MIB‐1 LI, *P* < 0.05 vs. V, N, and T) (Fig. 5a‐q, B), in contrast to N or TMZ single treatment (Fig. 5a‐g, l, B). As for apoptosis, while nimotuzumab monotherapy had no effect on apoptotic index (Fig. 5a‐h, i, C), TMZ treatment increased cell death by ~threefold (*P* < 0.05, vs. V), and NT combination treatment further enhanced apoptosis significantly by ~sixfold over controls (*P* < 0.05, vs. V, N) (Fig. 5a‐r, s, C). Similar findings were observed in subcutaneous xenografts after treatments (data not shown).

The influence of nimotuzumab on tumor angiogenesis, as it is required for tumor growth in vivo, was also determined. The tumor microvascular density, assessed by number of CD31‐stained microvessels, was only marginally reduced by N monotherapy. However, NT combination treatment which was active in tumor growth suppression (Fig. 3, 4) significantly lowered tumor vessel density (*P* < 0.01 vs. V, *P* < 0.05 vs. N). TMZ alone also decreased vessel density, but to a lesser extent (*P* < 0.01 vs. V) (Fig. 5a‐e, j, o, t, D).

### Resistance to the combination treatment is associated with decreased expression of mismatch repair proteins

**Figure 6:**
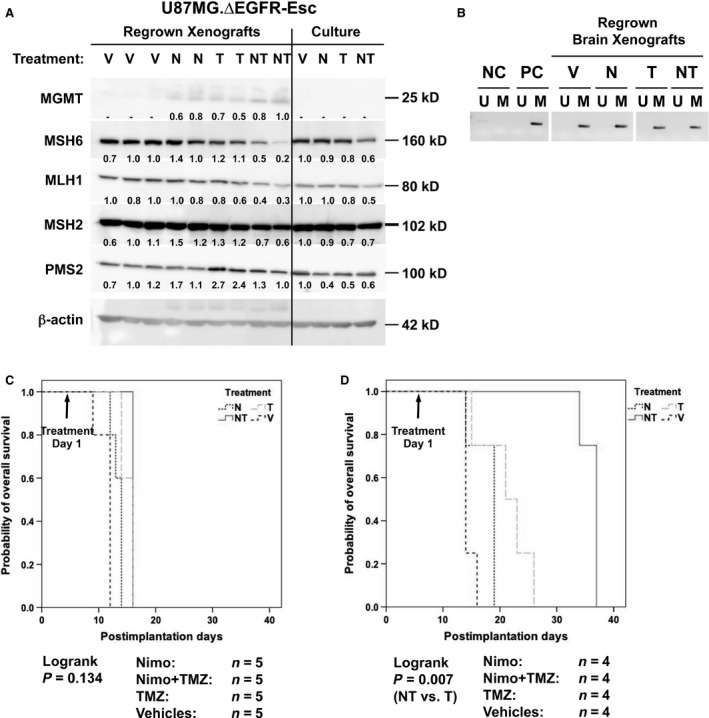
Expression and methylation status of *O* ^*6*^‐methylguanine‐DNA methyltransferase (MGMT) and mismatch repair (MMR)‐related molecules in post‐treatment recurred U87MG.∆EGFR escaper tumor cells (U87MG.∆EGFR escaper) and their treatment sensitivity in vivo. Nude mice bearing U87MG.∆EGFR xenografts were treated with either vehicles (Vehicles or V), nimotuzumab alone (Nimo or N), TMZ alone (TMZ or T), or combination with nimotuzumab and TMZ (Nimo+TMZ or NT) as in Fig. 3, and the tumors which had regrown (escapers) to a full size after the treatments were removed. Regrown tumors were put back to cell culture to re‐establish escaper cell lines. A. Western blot analysis of lysates from regrown xenografts and re‐established cultured cells (Culture) showing reduced expression of MSH6 and MLH1 by NT treatment. β‐actin blot was used as an internal control. Relative expression level of MMR proteins is shown below each lane, calculated as a ratio of that of control and standardized with actin expression. For MGMT, the highest expression in NT treatment is used as a control. Molecular sizes were shown at the right margin of the panels. B. Methylation‐specific PCR (MSP) for the *MGMT* promoter region showing the totally methylated pattern in all escaper xenografts. The presence of a PCR product in the *U Lane* signifies the presence of unmethylated *MGMT* promoter (undetectable in all samples), whereas a PCR product in the *M Lane* indicates the presence of methylated promoter. Normal peripheral blood leukocyte DNA (NC) was negative for methylation, whereas human genomic DNA with enzymatic methylation (PC) was used as a positive control for methylation. C, D. Abrogation of sensitivity to nimotuzumab/TMZ combination treatment in NT‐escaper intracerebral tumors. BALB/CA mice bearing intracerebral xenografts derived from U87MG.∆EGFR‐NT‐escaper (C), or U87MG.∆EGFR parental cells (D) were treated with vehicles (Vehicles or V), nimotuzumab alone (Nimo or N), TMZ alone (TMZ or T), or combination with nimotuzumab and TMZ (Nimo+TMZ or NT) as in Fig. 3. Survival curves for U87MG.∆EGFR parental intracerebral tumors were shown as a control for the treatment (*P* = 0.007, log‐rank test).

The NT combination treatment significantly suppressed growth of EGFRvIII‐overexpressing tumors and extended survival of mice with tumors. However, the treated tumors eventually recurred after the initial response in the absence of repeated treatments and finally killed the host animals, suggesting that regrown tumor cells may have had upfront or acquired resistance to the combination treatment. The expression levels of MGMT‐ and MMR‐related proteins in U87MG.∆EGFR xenografts which had recurred after each treatment type (U87MG.∆EGFR escaper) (Fig. 6A) were assessed. MGMT expression was undetectable in vehicle controls, whereas it was slightly detectable in the other treatments; the escapers of NT combination (NT escapers) showed the highest level. As for MMR proteins, the expression levels of both MSH6 and MLH1 were reduced in the NT escapers. After reculturing these escaper tumors in monolayer culture conditions, both MSH6 and MLH1 expression levels remained lower than those in other escaper cells derived from vehicles or single treatments. In contrast, MGMT expression was totally abrogated in recultured cells. Consistent with this finding, *MGMT* promoter methylation status remained fully methylated in all escapers, suggesting no selection of cells with unmethylated *MGMT* promoter after recurrence (Fig. 6B).

Whether the U87MG.∆EGFR‐NT‐escaper cells with lowered MSH6/MLH1 levels were still resistant to the combination treatment in vivo was determined. NT combination treatment no longer bestowed a survival benefit to mice carrying intracerebral U87MG.∆EGFR‐NT‐escaper xenografts over vehicle control, or each single treatment (Fig. 6C), while significantly extending the life span of mice with intracerebral U87MG.∆EGFR parental cells yet (*P* = 0.007, NT vs. T, log‐rank test) (Fig. 6D). Thus, NT‐escaper cells retained the resistant phenotype to the nimotuzumab‐enhanced TMZ antitumor effect, which might be associated with reduced MSH6/MLH1 expression.

## Discussion

The limited efficacy of the current standard of care for GBM 1 necessitates development of new therapeutic approaches, in particular targeting tumor‐specific molecules that are involved in tumor cell growth and maintenance. EGFR, especially EGFRvIII, has been nominated as a promising candidate as a therapeutic target in glioma because it is absent from normal cells and it has potent tumor promoting biological effects 5, 8, 9. This notion was previously tested using the small molecule EGFR inhibitor, AG1478, in human glioma U87MG cells overexpressing EGFRvIII, where strong inhibition of EGFRvIII phosphorylation resulted in sensitization of tumors to the cytotoxic agent cisplatin 9, 10.

Nimotuzumab has several unique characteristics compared with other anti‐EGFR mAbs and EGFR TKIs. It binds to EGFR at its extracellular domain III with 1/10‐ to 1/100‐fold lower affinity than cetuximab and panitumumab 26, 27, and this binding characteristics require cells expressing EGFR at a density of 10^4^ or 10^5^ receptors per cell or higher 28, and inhibit EGF binding while allowing EGFR to adopt its active conformation and a basal level of signaling 15. These properties illustrate a clear contrast with cetuximab which interacts strongly with the receptors even at low densities as in normal cells and suppresses their activation, leading to severe and problematic dose limiting adverse effects with cetuximab and other anti‐EGFR mAbs or TKIs.

In clinical studies, nimotuzumab had been used on the treatment of high‐grade malignancy astrocytic tumors in adult patients and children 18, 19, 21, 29, 30, 31. In adult Phase I/II study using nimotuzumab in combination with RT in 29 patients with anaplastic astrocytomas (AA) and GBM, an objective response rate of 37.9% was achieved, while stable disease was seen on 41.4% of the patients. Median overall survival (OS) was 22.2 months 21. In a Phase III, randomized, placebo‐controlled study using RT in combination with nimotuzumab or placebo in 41 patients with AA and 29 patients with GBM, the median OS was 17.8 months for the nimotuzumab and RT versus 12.6 months for RT 22. In another Phase III study, using RT and TMZ and nimotuzumab in 149 newly diagnosed GBM patients, the median OS in patients with residual tumor was 19.5 months for TMZ and RT and nimotuzumab versus 16.7 months for TMZ and RT 31. In children in a Phase II in 45 patients with relapsing or refractory progressive GBM, AA, or DIPG after primary, second, or third line of therapy, nimotuzumab was used single agent, 2 of 45 patients (4.4%) achieved partial response, and 15 (33.3%) achieved stable disease. The disease control rate was 37.8% and median OS of 4.7 months. In a Phase II study run in USA, Canada, and Israel, nimotuzumab was used as monotherapy in 44 children with relapsing DIPG. Nineteen patients completed 8 weeks of treatment: There were two partial responses (4.5%) and six stable diseases (13.6%). The disease control rate was of 18.2%, the median OS was 3.2 months, and there were two patients who lived 22 and 16 months from the start of nimotuzumab treatment 18. In a Phase III study on newly diagnosed children with DIPG, nimotuzumab was used in combination with external RT in 42 patients, the median progression‐free survival was 5.8 months, and the OS was 9.4 months. Two patients were alive 2 years after the start of treatment, and one patient is still stable and well 6 years after the primary treatment 19.

In this paper, we show that the anti‐EGFR mAb nimotuzumab inhibits EGFRvIII phosphorylation and can enhance TMZ‐induced growth suppression of tumors overexpressing EGFRvIII, and wtEGFR to a lesser extent, but not in tumors with a trace level of EGFR expression in vivo. It is for the first time to demonstrate that nimotuzumab affects oncogenic EGFRvIII.

In addition, there has been no clear clinical evidence that higher affinity leads to greater efficacy. Nimotuzumab thus may have an advantage for specifically targeting tumor cells with EGFR overexpression while sparing normal cells. It had been observed that the combination treatment with TMZ exerted enhanced antitumor effects in xenografts of U87MG.∆EGFR cells that have a EGFRvIII density of ~10^6^ receptors per cell 6, and lack of such synergy in tumors of U87MG with endogenous levels of EGFR. These results are consistent with the findings reported by Akashi et al.^32^ where nimotuzumab enhanced the antitumor effect of radiation in nonsmall cell lung cancer cell lines with high levels of surface EGFR expression, but not in those with low levels.

The antitumor efficacy in vivo was associated with marked dephosphorylation and also reduced expression levels of EGFRvIII (Fig. 2B, C), and decreased proliferation and increased apoptosis, consistent with previous studies 10, 17, 23. These effects, however, were only obtained when nimotuzumab was combined with TMZ, perhaps because the incomplete inhibition by nimotuzumab of EGFR signaling may lead to a decreased cytotoxic activity by itself 17. Alternatively, downregulation of EGFRvIII activation at both tyrosine phosphorylation and expression levels by the combination treatment may be important to suppress the signaling from EGFR that contributes to growth and survival benefits for glioma cells in vivo. The underlying mechanisms by which addition of TMZ leads to reduced expression, for instance, facilitating internalization and degradation, or inhibiting synthesis, need to be elucidated.

Nimotuzumab had also shown to downregulate vascular endothelial growth factor (VEGF) expression and to inhibit angiogenesis after treatment in A431 squamous cell carcinoma xenografts 16, as well as in U87MG glioma xenografts in combination with radiotherapy 17. A close relationship between EGFR and VEGF has been postulated in angiogenesis, and indeed, EGFRvIII has shown to promote glioma angiogenesis 8. Accordingly, it had been shown on this paper that the vessel density in xenografts significantly diminished upon NT combination treatment as compared to controls (Fig. 5), and this might account in part for why nimotuzumab did not enhance TMZ‐induced cytotoxicity in EGFR‐overexpressing glioma cells in vitro.

Another interesting finding is that nimotuzumab appears to affect in vivo tumor growth in combination with TMZ to a higher extent in tumors with EGFRvIII overexpression than in those with overexpressed wtEGFR (Fig. 3, 4). Although the epitope for nimotuzumab binding to EGFR is located in its domain III which is preserved in the EGFRvIII mutant lacking a part of domains I and II 3, 15, it is at present unclear whether the conformation of this domain remains the same between the receptors, and whether nimotuzumab can bind to EGFRvIII at a similar stringency or higher to wtEGFR, which might be suggested by the differential inhibition profile of phosphorylation of EGFR and Akt by nimotuzumab at low doses (Fig. 1). Difference in receptor tyrosine phosphorylation level and endocytic rate upon activation between these receptors may also account for this profile (only ~12% activation in EGFRvIII compared with wtEGFR) 6. Although EGFRvIII has not been documented in all GBMs (ranging from 15 to 50%), the impact of EGFRvIII on aggressive behaviors of malignant glioma has been shown to be more prominent than wtEGFR 6, and thus it is worth further investigating its differential binding affinity to these receptors and resulting changes in downstream signaling to determine whether nimotuzumab has selected activity on EGFRvIII, as does mAb 806 23.

Although MGMT plays a direct role in regulating TMZ sensitivity, reduced MMR expression but not change in *MGMT* status has frequently been detected in recurrent GBMs, suggesting its association with acquired resistance to TMZ 33. Indeed, it had been found that the NT escapers, no longer sensitive to NT combination, expressed lower levels of both MSH6 and MLH1 while retaining methylated *MGMT* promoter. Furthermore, these results suggest that the antitumor effects of the NT combination treatment may be largely attributable to TMZ‐induced cytotoxicity and that suppression of EGFR‐mediated signaling by nimotuzumab may function as a co‐activator of TMZ action. As only a limited number of escaper cell strains were used, further studies including multiple cell strains as well as gene expression profiling may be considered to elucidate other mechanisms involved in the resistance such as changes in expression level of key molecules in EGFR signaling.

TMZ can effectively penetrate the blood–brain barrier (BBB) 34, while macromolecules like mAbs show limited penetration to the intact brain, although they may cross BBB in clinically relevant concentrations in tumors with contrast enhancement, as seen in high‐grade gliomas, which is indicative of a disturbed BBB 23. To supplement this issue, convection‐enhanced delivery may help delivery of BBB‐noncrossing therapeutic agents and has been exploited to localize mAbs to tumor‐infiltrating regions in the brain 35, 36.

In conclusions, the present study has demonstrated that nimotuzumab enhanced the antitumor efficacy of TMZ in human glioma cells overexpressing EGFR, especially the most common mutant EGFRvIII, in vivo. Recurrent tumor cells acquired resistance to the nimotuzumab and TMZ combination treatment, which was associated with reduced expression of the mismatch repair proteins, MSH6 and MLH1. These results provide a basis for the selection of patients with GBM by EGFR status who may well benefit from the nimotuzumab and TMZ combination therapy in the future clinical trials.

## Conflict of interest

Dr. Motoo Nagane has received research funding from Daiichi Sankyo Co., Ltd. and honoraria from Chugai Pharmaceutical Co., Ltd. No other conflict of interests were declared.

## References

[1] Stupp, R. , W. P. Mason , M. J. van den Bent , M. Weller , B. Fisher , M. J. Taphoorn , et al. 2005 Radiotherapy plus concomitant and adjuvant temozolomide for glioblastoma. N. Engl. J. Med. 352:987–996. 15758009 10.1056/NEJMoa043330

[2] Network, T. C. G. A. R. . 2008 Comprehensive genomic characterization defines human glioblastoma genes and core pathways. Nature 455:1061–1068. 18772890 10.1038/nature07385 PMC2671642

[3] Ekstrand, A. J. , N. Sugawa , C. D. James , and V. P. Collins . 1992 Amplified and rearranged epidermal growth factor receptor genes in human glioblastomas reveal deletions of sequences encoding portions of the N‐ and/or C‐terminal tails. Proc. Natl. Acad. Sci. U. S. A. 89:4309–4313. 1584765 10.1073/pnas.89.10.4309 PMC49071

[4] Schlegel, J. , A. Merdes , G. Stumm , F. K. Albert , M. Forsting , N. Hynes , et al. 1994 Amplification of the epidermal‐growth‐factor‐receptor gene correlates with different growth behaviour in human glioblastoma. Int. J. Cancer 56:72–77. 8262681 10.1002/ijc.2910560114

[5] Nishikawa, R. , X. D. Ji , R. C. Harmon , C. S. Lazar , G. N. Gill , W. K. Cavenee , et al. 1994 A mutant epidermal growth factor receptor common in human glioma confers enhanced tumorigenicity. Proc. Natl. Acad. Sci. U. S. A. 91:7727–7731. 8052651 10.1073/pnas.91.16.7727 PMC44475

[6] Huang, H. S. , M. Nagane , C. K. Klingbeil , H. Lin , R. Nishikawa , X. D. Ji , et al. 1997 The enhanced tumorigenic activity of a mutant epidermal growth factor receptor common in human cancers is mediated by threshold levels of constitutive tyrosine phosphorylation and unattenuated signaling. J. Biol. Chem. 272:2927–2935. 9006938 10.1074/jbc.272.5.2927

[7] Nagane, M. , F. Coufal , H. Lin , O. Bögler , W. K. Cavenee , and H. J. S. Huang . 1996 A common mutant epidermal growth factor receptor confers enhanced tumorigenicity on human glioblastoma cells by increasing proliferation and reducing apoptosis. Cancer Res. 56:5079–5086. 8895767

[8] Bon avia, R. , M. M. Inda , S. Vandenberg , S. Y. Cheng , M. Nagane , P. Hadwiger , et al. 2012 EGFRvIII promotes glioma angiogenesis and growth through the NF‐kappaB, interleukin‐8 pathway. Oncogene 31:4054–4066. 22139077 10.1038/onc.2011.563 PMC3537826

[9] Nagane, M. , A. Levitzki , A. Gazit , W. K. Cavenee , and H. J. Huang . 1998 Drug resistance of human glioblastoma cells conferred by a tumor‐specific mutant epidermal growth factor receptor through modulation of Bcl‐XL and caspase‐3‐like proteases. Proc Natl Acad Sci U S A. 95:5724–5729. 9576951 10.1073/pnas.95.10.5724 PMC20446

[10] Nagane, M. , Y. Narita , K. Mishima , A. Levitzki , A. W. Burgess , W. K. Cavenee , et al. 2001 Human glioblastoma xenografts overexpressing a tumor‐specific mutant epidermal growth factor receptor sensitized to cisplatin by the AG1478 tyrosine kinase inhibitor. J. Neurosurg. 95:472–479. 11565870 10.3171/jns.2001.95.3.0472

[11] Brandes, A. A. , E. Franceschi , A. Tosoni , M. E. Hegi , and R. Stupp . 2008 Epidermal growth factor receptor inhibitors in neuro‐oncology: hopes and disappointments. Clin. Cancer Res. 14:957–960. 18281526 10.1158/1078-0432.CCR-07-1810

[12] Bonner, J. A. , P. M. Harari , J. Giralt , N. Azarnia , D. M. Shin , R. B. Cohen , et al. 2006 Radiotherapy plus cetuximab for squamous‐cell carcinoma of the head and neck. N. Engl. J. Med. 354:567–578. 16467544 10.1056/NEJMoa053422

[13] Eller, J. L. , S. L. Longo , M. M. Kyle , D. Bassano , D. J. Hicklin , and G. W. Canute . 2005 Anti‐epidermal growth factor receptor monoclonal antibody cetuximab augments radiation effects in glioblastoma multiforme in vitro and in vivo. Neurosurgery 56:155–162; discussion 62. 15617598 10.1227/01.neu.0000145865.25689.55

[14] Neyns, B. , J. Sadones , E. Joosens , F. Bouttens , L. Verbeke , J. F. Baurain , et al. 2009 Stratified phase II trial of cetuximab in patients with recurrent high‐grade glioma. Ann. Oncol. 20:1596–1603. 19491283 10.1093/annonc/mdp032

[15] Talavera, A. , R. Friemann , S. Gomez‐Puerta , C. Martinez‐Fleites , G. Garrido , A. Rabasa , et al. 2009 Nimotuzumab, an antitumor antibody that targets the epidermal growth factor receptor, blocks ligand binding while permitting the active receptor conformation. Cancer Res. 69:5851–5859. 19584289 10.1158/0008-5472.CAN-08-4518

[16] Crombet‐Ramos, T. , J. Rak , R. Perez , and A. Viloria‐Petit . 2002 Antiproliferative, antiangiogenic and proapoptotic activity of h‐R3: a humanized anti‐EGFR antibody. Int. J. Cancer 101:567–575. 12237899 10.1002/ijc.10647

[17] Diaz Miqueli, A. , J. Rolff , M. Lemm , I. Fichtner , R. Perez , and E. Montero . 2009 Radiosensitisation of U87MG brain tumours by anti‐epidermal growth factor receptor monoclonal antibodies. Br. J. Cancer 100:950–958. 19293809 10.1038/sj.bjc.6604943 PMC2661790

[18] Bartels, U. , J. Wolff , L. Gore , I. Dunkel , S. Gilheeney , J. Allen , et al. 2014 Phase 2 study of safety and efficacy of nimotuzumab in pediatric patients with progressive diffuse intrinsic pontine glioma. Neuro. Oncol. 16:1554–1559. 24847085 10.1093/neuonc/nou091 PMC4201068

[19] Bode, U. , M. Massimino , F. Bach , M. Zimmermann , E. Khuhlaeva , M. Westphal , et al. 2012 Nimotuzumab treatment of malignant gliomas. Expert. Opin. Biol. Ther. 12:1649–1659. 23043252 10.1517/14712598.2012.733367

[20] Boland, W. K. , and G. Bebb . 2009 Nimotuzumab: a novel anti‐EGFR monoclonal antibody that retains anti‐EGFR activity while minimizing skin toxicity. Expert. Opin. Biol. Ther. 9:1199–1206. 19624281 10.1517/14712590903110709

[21] Ramos, T. C. , J. Figueredo , M. Catala , S. Gonzalez , J. C. Selva , T. M. Cruz , et al. 2006 Treatment of high‐grade glioma patients with the humanized anti‐epidermal growth factor receptor (EGFR) antibody h‐R3: report from a phase I/II trial. Cancer Biol. Ther. 5:375–379. 16575203 10.4161/cbt.5.4.2522

[22] Solomon, M. T. , J. C. Selva , J. Figueredo , J. Vaquer , C. Toledo , N. Quintanal , et al. 2013 Radiotherapy plus nimotuzumab or placebo in the treatment of high grade glioma patients: results from a randomized, double blind trial. BMC Cancer 13:299. 23782513 10.1186/1471-2407-13-299 PMC3691625

[23] Mishima, K. , T. G. Johns , R. B. Luwor , A. M. Scott , E. Stockert , A. A. Jungbluth , et al. 2001 Growth suppression of intracranial xenografted glioblastomas overexpressing mutant epidermal growth factor receptors by systemic administration of monoclonal antibody (mAb) 806, a novel monoclonal antibody directed to the receptor. Cancer Res. 61:5349–5354. 11454673

[24] Tanaka, G. , and Y. Nakazato . 2004 Automatic quantification of the MIB‐1 immunoreactivity in brain tumors Pp. 15–19 in Watanabe K, ed. Developments in Neuroscience Proceedings on the 3rd International Mt Bandai Symposium for Neuroscience and the 4th Pan‐Pacific Neurosurgery Congress. International Congress Series. 1259. Elsevier B.V, Amsterdam.

[25] Esteller, M. , S. R. Hamilton , P. C. Burger , S. B. Baylin , and J. G. Herman . 1999 Inactivation of the DNA repair gene O6‐methylguanine‐DNA methyltransferase by promoter hypermethylation is a common event in primary human neoplasia. Cancer Res. 59:793–797. 10029064

[26] Crombet, T. , M. Osorio , T. Cruz , C. Roca , R. del Castillo , R. Mon , et al. 2004 Use of the humanized anti‐epidermal growth factor receptor monoclonal antibody h‐R3 in combination with radiotherapy in the treatment of locally advanced head and neck cancer patients. J. Clin. Oncol. 22:1646–1654. 15117987 10.1200/JCO.2004.03.089

[27] Crombet, T. , L. Torres , E. Neninger , M. Catala , M. E. Solano , A. Perera , et al. 2003 Pharmacological evaluation of humanized anti‐epidermal growth factor receptor, monoclonal antibody h‐R3, in patients with advanced epithelial‐derived cancer. J. Immunother. 26:139–148. 12616105 10.1097/00002371-200303000-00006

[28] Garrido, G. , I. A. Tikhomirov , A. Rabasa , E. Yang , E. Gracia , N. Iznaga , et al. 2011 Bivalent binding by intermediate affinity of nimotuzumab: a contribution to explain antibody clinical profile. Cancer Biol. Ther. 11:373–382. 21150278 10.4161/cbt.11.4.14097

[29] MacDonald, T. J. , D. Aguilera , and C. M. Kramm . 2011 Treatment of high‐grade glioma in children and adolescents. Neuro. Oncol. 13:1049–1058. 21784756 10.1093/neuonc/nor092 PMC3177659

[30] Massimino, M. , U. Bode , V. Biassoni , and G. Fleischhack . 2011 Nimotuzumab for pediatric diffuse intrinsic pontine gliomas. Expert. Opin. Biol. Ther. 11:247–256. 21171927 10.1517/14712598.2011.546341

[31] Westphal, M. , O. Heese , J. P. Steinbach , O. Schnell , G. Schackert , M. Mehdorn , et al. 2015 A randomised, open label phase III trial with nimotuzumab, an anti‐epidermal growth factor receptor monoclonal antibody in the treatment of newly diagnosed adult glioblastoma. Eur. J. Cancer 51:522–532. 25616647 10.1016/j.ejca.2014.12.019

[32] Akashi, Y. , I. Okamoto , T. Iwasa , T. Yoshida , M. Suzuki , E. Hatashita , et al. 2008 Enhancement of the antitumor activity of ionising radiation by nimotuzumab, a humanised monoclonal antibody to the epidermal growth factor receptor, in non‐small cell lung cancer cell lines of differing epidermal growth factor receptor status. Br. J. Cancer 98:749–755. 18253126 10.1038/sj.bjc.6604222 PMC2259177

[33] Felsberg, J. , N. Thon , S. Eigenbrod , B. Hentschel , M. C. Sabel , M. Westphal , et al. 2011 Promoter methylation and expression of MGMT and the DNA mismatch repair genes MLH1, MSH2, MSH6 and PMS2 in paired primary and recurrent glioblastomas. Int. J. Cancer 129:659–670. 21425258 10.1002/ijc.26083

[34] Danson, S. J. , and M. R. Middleton . 2001 Temozolomide: a novel oral alkylating agent. Expert Rev. Anticancer Ther. 1:13–19. 12113120 10.1586/14737140.1.1.13

[35] Kunwar, S. , S. Chang , M. Westphal , M. Vogelbaum , J. Sampson , G. Barnett , et al. 2010 Phase III randomized trial of CED of IL13‐PE38QQR vs Gliadel wafers for recurrent glioblastoma. Neuro Oncol. 12:871–881. 20511192 10.1093/neuonc/nop054 PMC2940677

[36] Bidros, D. S. , and M. A. Vogelbaum . 2009 Novel drug delivery strategies in neuro‐oncology. Neurotherapeutics: the journal of the American Society for Experimental. Neurotherapeutics 6:539–546. 19560743 10.1016/j.nurt.2009.04.004 PMC5084189

